# The *ARGOS* gene family functions in a negative feedback loop to desensitize plants to ethylene

**DOI:** 10.1186/s12870-015-0554-x

**Published:** 2015-06-24

**Authors:** Muneeza Iqbal Rai, Xiaomin Wang, Derek M. Thibault, Hyo Jung Kim, Matthew M. Bombyk, Brad M. Binder, Samina N. Shakeel, G. Eric Schaller

**Affiliations:** Department of Biological Sciences, Dartmouth College, Hanover, NH 03755 USA; Department of Biochemistry, Quaid-i-azam University, Islamabad, 45320 Pakistan; Department of Biochemistry and Cellular & Molecular Biology, University of Tennessee, Knoxville, TN 37996 USA

**Keywords:** Ethylene, Desensitization, Ethylene receptor, Endoplasmic reticulum, Auxin, Arabidopsis

## Abstract

**Background:**

Ethylene plays critical roles in plant growth and development, including the regulation of cell expansion, senescence, and the response to biotic and abiotic stresses. Elements of the initial signal transduction pathway have been determined, but we are still defining regulatory mechanisms by which the sensitivity of plants to ethylene is modulated.

**Results:**

We report here that members of the *ARGOS* gene family of Arabidopsis, previously implicated in the regulation of plant growth and biomass, function as negative feedback regulators of ethylene signaling. Expression of all four members of the *ARGOS* family is induced by ethylene, but this induction is blocked in ethylene-insensitive mutants. The dose dependence for ethylene induction varies among the *ARGOS* family members, suggesting that they could modulate responses across a range of ethylene concentrations. GFP-fusions of ARGOS and ARL localize to the endoplasmic reticulum, the same subcellular location as the ethylene receptors and other initial components of the ethylene signaling pathway. Seedlings with increased expression of *ARGOS* family members exhibit reduced ethylene sensitivity based on physiological and molecular responses.

**Conclusions:**

These results support a model in which the *ARGOS* gene family functions as part of a negative feedback circuit to desensitize the plant to ethylene, thereby expanding the range of ethylene concentrations to which the plant can respond. These results also indicate that the effects of the *ARGOS* gene family on plant growth and biomass are mediated through effects on ethylene signal transduction.

**Electronic supplementary material:**

The online version of this article (doi:10.1186/s12870-015-0554-x) contains supplementary material, which is available to authorized users.

## Background

The gaseous hormone ethylene plays critical roles in plant growth and development, including the regulation of cell expansion, senescence, and the response to biotic and abiotic stresses [1]. Key elements in the ethylene signaling pathway have been identified through the characterization of ethylene insensitive and constitutive ethylene response mutants of Arabidopsis, double mutant analysis then allowing for the ordering of these elements into a signaling pathway [2, 3]. These signaling elements are, in order, an ethylene receptor family related to the histidine kinases of prokaryotes, the Raf-like kinase CTR1, the Nramp-like protein EIN2, and the EIN3 family of transcription factors. This signal transduction pathway transduces the ethylene signal from the membrane-bound receptors to the nucleus, where the EIN3 transcription factors mediate the characteristic transcriptional response to ethylene.

Interestingly, the ethylene receptors as well as the initial signaling elements in the pathway are predominantly localized to the endoplasmic reticulum (ER) [4]. The ER is an unusual location for a hormone receptor but is compatible with the ready diffusion of ethylene in aqueous and lipid environments. The proximity of the receptors, CTR1, and EIN2 at the ER would facilitate transmission of the ethylene signal, which is thought to operate according to the following model. In the absence of ethylene, the receptors activate the Raf-like kinase CTR1 [5]. The direct phosphorylation target of CTR1 is EIN2, which is maintained in an inactive state when phosphorylated by CTR1, thereby resulting in a suppression of the ethylene response [6–8]. Upon ethylene binding, the receptors inactivate CTR1, thereby relieving suppression on the downstream signaling elements. As a result, EIN2 is proteolytically processed such that a C-terminal domain is released to migrate to the nucleus, where it either directly or indirectly activates the transcription factors *EIN3* and *EIN3 like1* (*EIL1*) to initiate the transcriptional response to ethylene [6–8].

Arabidopsis seedlings can sense and respond to a tremendous range of ethylene concentrations, spanning over six orders of magnitude [9, 10]. Diverse mechanisms exist by which output from the ethylene signaling pathway can be modulated to regulate the plant’s ethylene sensitivity [11]. These include transcriptional regulation and proteasome-mediated degradation of key signaling elements [12–17], clustering of receptors [18–20], and interactions of pathway elements with auxiliary proteins such as the RTE1/GR family [21, 22]. In addition, various genes have been identified as modulating the ethylene response based on a genetic screen for enhanced ethylene sensitivity [23–27].

We report here that the *AUXIN REGULATED GENE INVOLVED IN ORGAN SIZE* (*ARGOS*) gene family functions to regulate output by the ethylene signal transduction pathway. *ARGOS* is the founding member of a four-member family of proteins, and its expression, as indicated by its name, is induced by auxin [28]. *ARGOS* is proposed to be a regulator of plant growth and biomass because overexpression increases organ size and antisense decreases organ size [28]. Other members of this family (*ARL*, *OSR1*, and *OSR2*) yield similar mutant phenotypes [29–31], consistent with an overlapping function in the control of plant growth. As reported here, our data indicate that a primary function of the ARGOS family is to control ethylene signaling, with the ARGOS family members functioning as negative feedback mediators to desensitize the plant to ethylene, a role that facilitates the ethylene adaptation response of plants. This function in the regulation of ethylene signaling may itself account for the majority of the phenotypes associated with mutations in the *ARGOS* family.

## Results

### Ethylene-dependent expression of the *ARGOS* gene family

The primary success to date in identifying elements of the ethylene signal transduction pathway (e.g. ETR1, EIN2, CTR1, and EIN3) has come through the employment of genetic approaches [2, 3]. We pursued an alternative approach to identify new ethylene signaling elements, based on the hypothesis that many signaling pathways induce the production of negative regulators for the pathway, a precedent in the ethylene pathway being the negative regulator RTE1 [22]. To this end, we examined microarray data for genes that are (1) induced by ethylene or the ethylene precursor 1-aminocyclopropane-1-carboxylic acid (ACC) and (2) encode proteins with predicted transmembrane domain(s), because these proteins could potentially be targeted to the endoplasmic reticulum where the initial signaling elements in the ethylene pathway are localized [4]. By this process we identified members of the *ARGOS* family (*ARGOS*, *ARL*, and *OSR1*), which have been previously reported to regulate organ size, with *OSR1* also identified as a gene induced in response to ACC [28–30]. The fourth member of the family (*OSR2*) is not present on the Affymetrix ATH1 genome array. The phylogenetic relationship between members of the ARGOS family is given in Additional file 1.

**Fig. 1:**
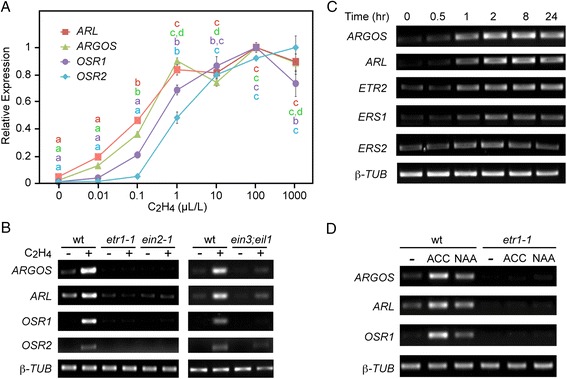
Ethylene induction of *ARGOS* family members. **a** Ethylene dose-dependency for induction varies among family members. Green seedlings were treated for 2 h in the presence or absence of the indicated ethylene concentrations, RNA isolated, and gene expression determined by qRT-PCR based on three biological replicates. Expression is shown relative to the maximal induction observed for each gene. Significant dose-dependent differences in expression for each gene are based on an analysis of variance applying Bonferroni correction post-test comparisons (p < 0.05); designations with the same letter exhibit no significant difference. Error bars indicate SE; error bars not shown if smaller than symbol. **b** Effect of ethylene-insensitive mutations on gene induction. Induction was examined in wild-type (wt) and the ethylene-insensitive mutants *etr1-1, ein2-1*, and *ein3;eil1* using 2-h treatments of green seedlings in the presence or absence of 10 μL L^−1^ ethylene. Gene expression was determined by semi-quantitative RT-PCR. **c** Time course for ethylene induction of *ARGOS* and *ARL*, as compared to the receptors *ETR2*, *ERS1*, and *ERS2*. Green seedlings were treated for indicated times with 10 μL L^−1^ ethylene and gene expression determined by semi-quantitative RT-PCR. **d** Auxin induction of ARGOS family members is ethylene-dependent. Wild-type or *etr1-1* green seedlings were treated for 3 h in the absence or presence of 50 μM ACC or 5 μM NAA. Gene expression was determined by semi-quantitative RT-PCR

All four members of the *ARGOS* family are induced by exogenous ethylene (Fig. 1a), but the dose dependence for ethylene-induction varies among the family members, induction of the closely related *ARGOS* and *ARL* being most sensitive to ethylene. Induction is blocked in the ethylene-insensitive mutants *etr1-1* and *ein2-1,* and reduced in *ein3;eil1* (which exhibits partial ethylene insensitivity) (Fig. 1b), demonstrating that expression is regulated through the well-characterized ethylene-signaling pathway [32, 33]. Time-course analysis indicated that both *ARGOS* and *ARL* are rapidly induced by ethylene, induction paralleling that of the ethylene receptor genes *ETR2*, *ERS1*, and *ERS2* (Fig. 1c).

**Fig. 2:**
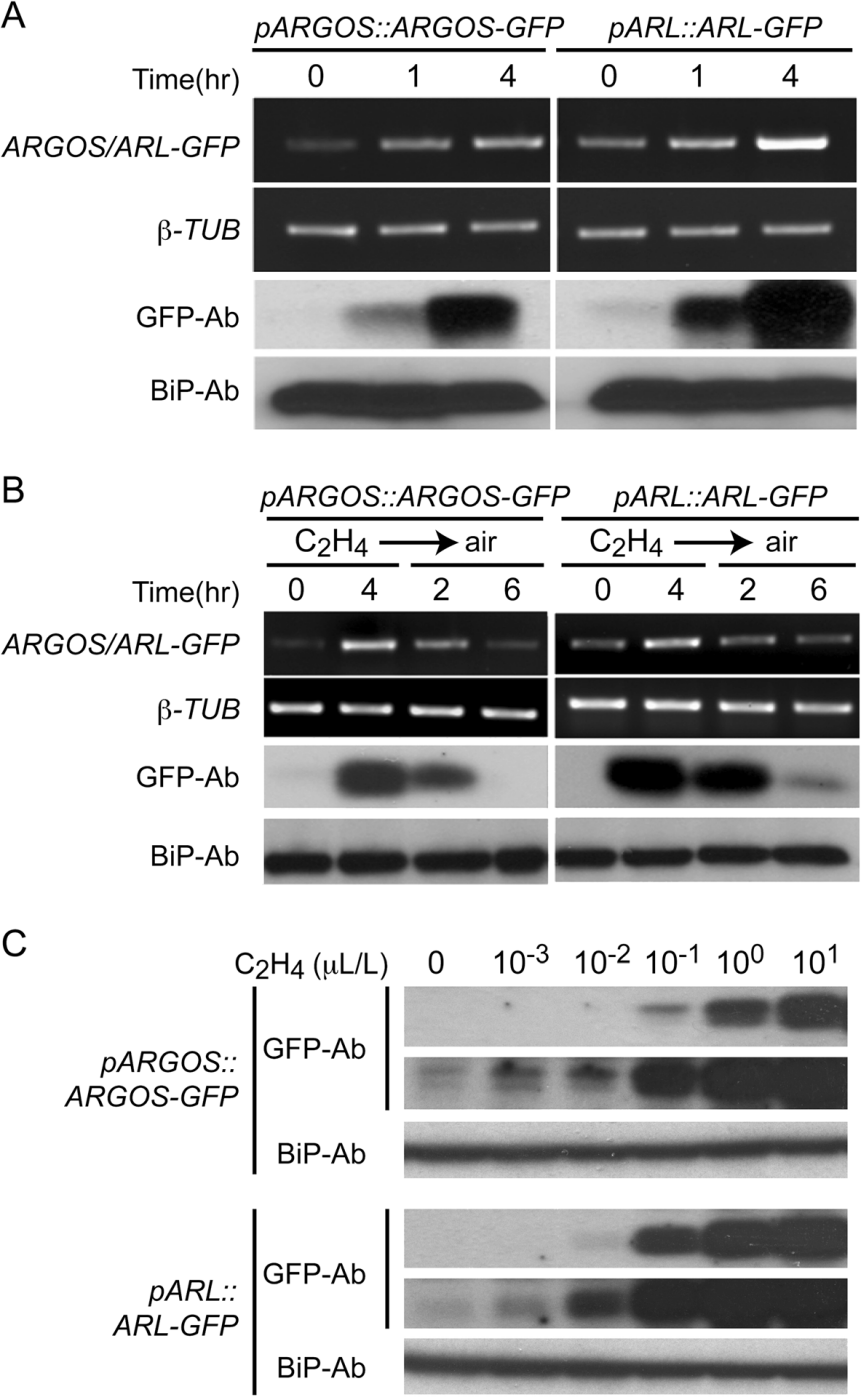
Ethylene-dependent regulation of ARGOS and ARL protein levels. GFP-tagged versions of *ARGOS* and *ARL*, driven by their native promoters, were stably expressed in Arabidopsis. Transcript levels for the *ARGOS-GFP* and *ARL-GFP* fusions were determined by RT-PCR, with β-tubulin as a control. Protein levels of the GFP fusions were determined by immunoblot analysis using an anti-GFP antibody, with BiP detected by an anti-BiP antibody as a loading control. **a** Time course for mRNA and protein induction of *ARGOS-GFP* and *ARL-GFP* by ethylene. Seedlings were treated with 10 μL L^−1^ for the indicated times. **b** Reduction in mRNA and protein levels following removal of ethylene. Expression of *ARGOS-GFP* and *ARL-GFP* was induced by ethylene treatment for 4 h, then the ethylene removed, and the kinetics for reduction followed at the RNA and protein levels. **c** Ethylene dose dependence for induction of ARGOS-GFP and ARL-GFP protein. Green seedlings were treated with the indicated ethylene concentrations for 4 h. Two immunoblot exposures are shown for the GFP fusions to allow for visualization of induction at low and high ethylene concentrations

To determine if the ethylene-induced changes in expression are reflected at the protein level we employed GFP-fusions to ARGOS and ARL driven from their native promoters (Fig. 2). In response to exogenous ethylene, both the ARGOS-GFP and ARL-GFP proteins are strongly induced, the level of induction being even greater than that observed for the transcripts (Fig. 2a). Upon removal of ethylene, transcript and protein levels for ARGOS-GFP and ARL-GFP rapidly drop, demonstrating tight control of expression by ethylene (Fig. 2b). Dose response analysis demonstrated that their protein induction is very responsive to ethylene, induction being detected in response to 0.001 μL L^−1^ ethylene (Fig. 2c).

Public microarray data indicates that only ethylene (as the ethylene precursor ACC) and auxin (indole-3-acetic acid; IAA) consistently induce expression of *ARGOS* gene family members, ACC demonstrating a stronger effect than IAA (Additional file 2). Members of the *ARGOS* family were previously reported to be induced by auxin [28, 30]. However auxin stimulates ethylene biosynthesis [1, 34, 35], raising the possibility that the effect of auxin on the induction of *ARGOS* gene family members might operate indirectly through the ethylene signaling pathway. To test this hypothesis, we examined the effect the ethylene-insensitive mutant *etr1-1* on the ability of auxin to induce expression of *ARGOS*, *ARL*, and *OSR1* (Fig. 1d). Auxin (1-naphthaleneacetic acid; NAA) induced expression of all three genes, but this effect was eliminated by the ethylene-insensitive mutant *etr1-1*. These results indicate that auxin induction of the *ARGOS* gene family is dependent on the ethylene signaling pathway.

### ARGOS family proteins are membrane-associated and localize to the endoplasmic reticulum

**Fig. 3:**
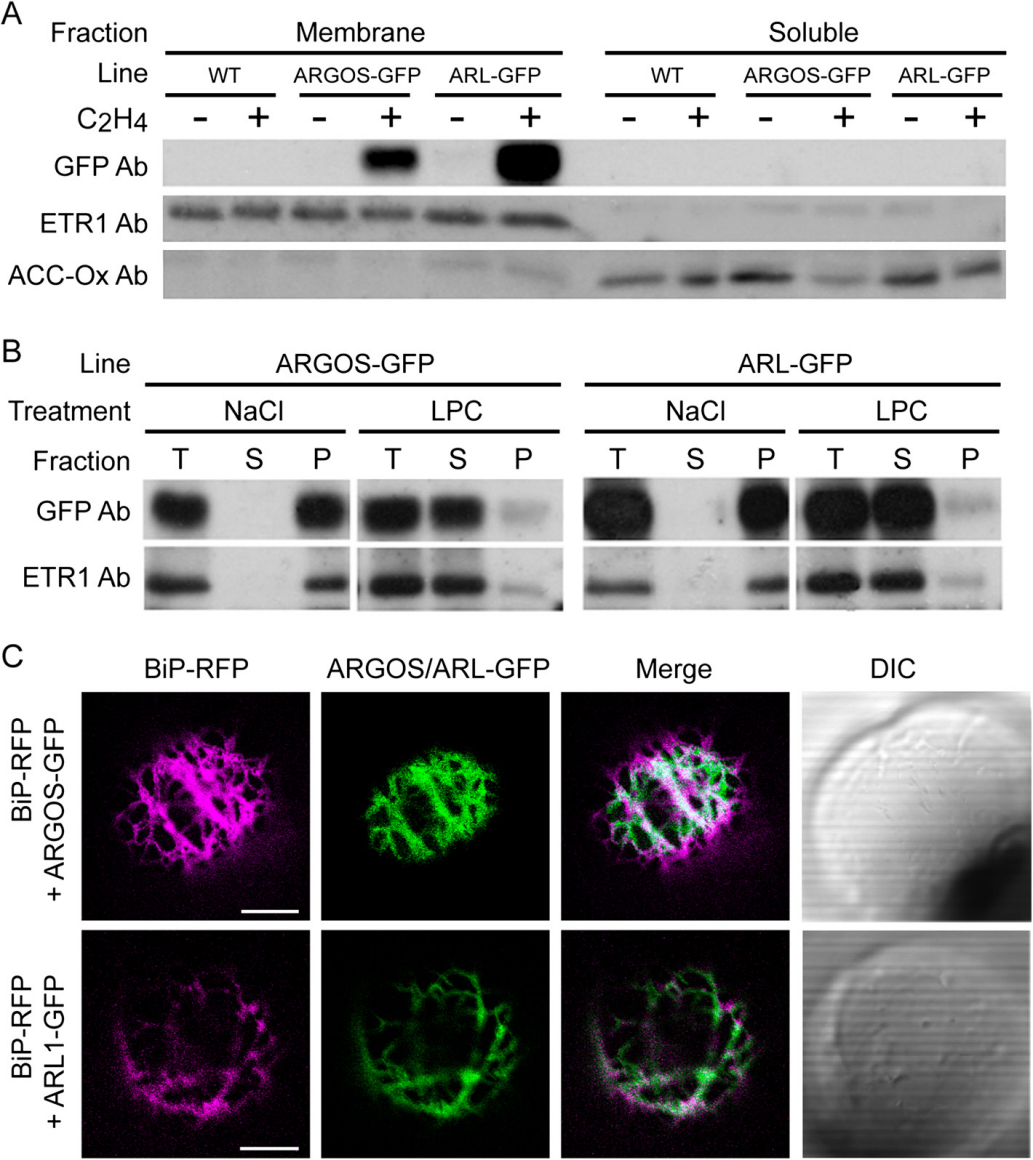
ARGOS and ARL are membrane-associated proteins localized to the ER. **a** Membrane association of ARGOS-GFP and ARL-GFP. Membrane and soluble fractions were isolated from green seedlings following 4-h treatment in the absence or presence of 10 μL L^−1^ ethylene. Immunoblot analysis was performed with anti-GFP antibody to detect the ARGOS-GFP and ARL-GFP. Immunological detection of the ethylene receptor ETR1 and ACC-oxidase served as markers for the membrane and the soluble fractions, respectively. **b** Strong association of ARGOS and ARL with membranes. Microsomal membranes were treated with 0.5 M NaCl or 0.5% (w/v) lysophosphatidylcholine (LPC). The different lanes represent the total membranes prior to centrifugation (T), and from the soluble (S) and (P) fractions after centrifugation. The relative amounts of ARGOS-GFP, ARL-GFP, and ETR1 were determined by immunoblot analysis, the membrane protein ETR1 serving as an internal control for solubilization. **c** ARGOS and ARL localize to the ER. Protoplasts were transfected with either ARGOS-GFP or ARL-GFP (green), along with the ER-marker BiP-RFP (magenta), and visualized by confocal microscopy. Regions of overlap are indicated by white on the merged image. DIC images of protoplasts are also shown. Focused region is on the cortical ER underlying the plasma membrane. Scale bars = 10 μm

Members of the ARGOS family contain two predicted transmembrane domains (Additional file 1). Prior analysis has suggested that members of the ARGOS family might be localized to nucleus, cytoplasm, plasma membrane and/or endoplasmic reticulum [28–31]. We found that the green-fluorescent protein (GFP) fusions of ARGOS and ARL are membrane-localized based on fractionation of transgenic plant lines into microsomal and soluble fractions (Fig. 3a). Furthermore, the fusion proteins are resistant to extraction from the membranes by sodium chloride, but can be solubilized from membranes when treated with the detergent lysophosphatidylcholine (Fig. 3b). In this respect, they are similar to the transmembrane ethylene-receptor ETR1 [36], consistent with ARGOS and ARL being transmembrane proteins. Although readily detectable by immunoblot analysis, we could not detect ARGOS-GFP and ARL-GFP based on their GFP fluorescence in these stable transgenic lines. Therefore, to determine the subcellular localization of ARGOS and ARL, we transiently expressed the GFP fusions in Arabidopsis protoplasts (Fig. 3c). The resulting fluorescence co-localized with the ER-marker BiP-RFP. Furthermore, the fluorescence in the region underlying the plasma membrane exhibited the distinctive reticulate network appearance found with the cortical ER (Fig. 3c). No localization to the plasma membrane itself was detected. Thus, both the co-localization with BiP and morphological features of the membrane network support ER localization for ARGOS and ARL, consistent with results reported by Feng et al. [29].

### The *ARGOS* family regulates ethylene sensitivity of seedlings

**Fig. 4:**
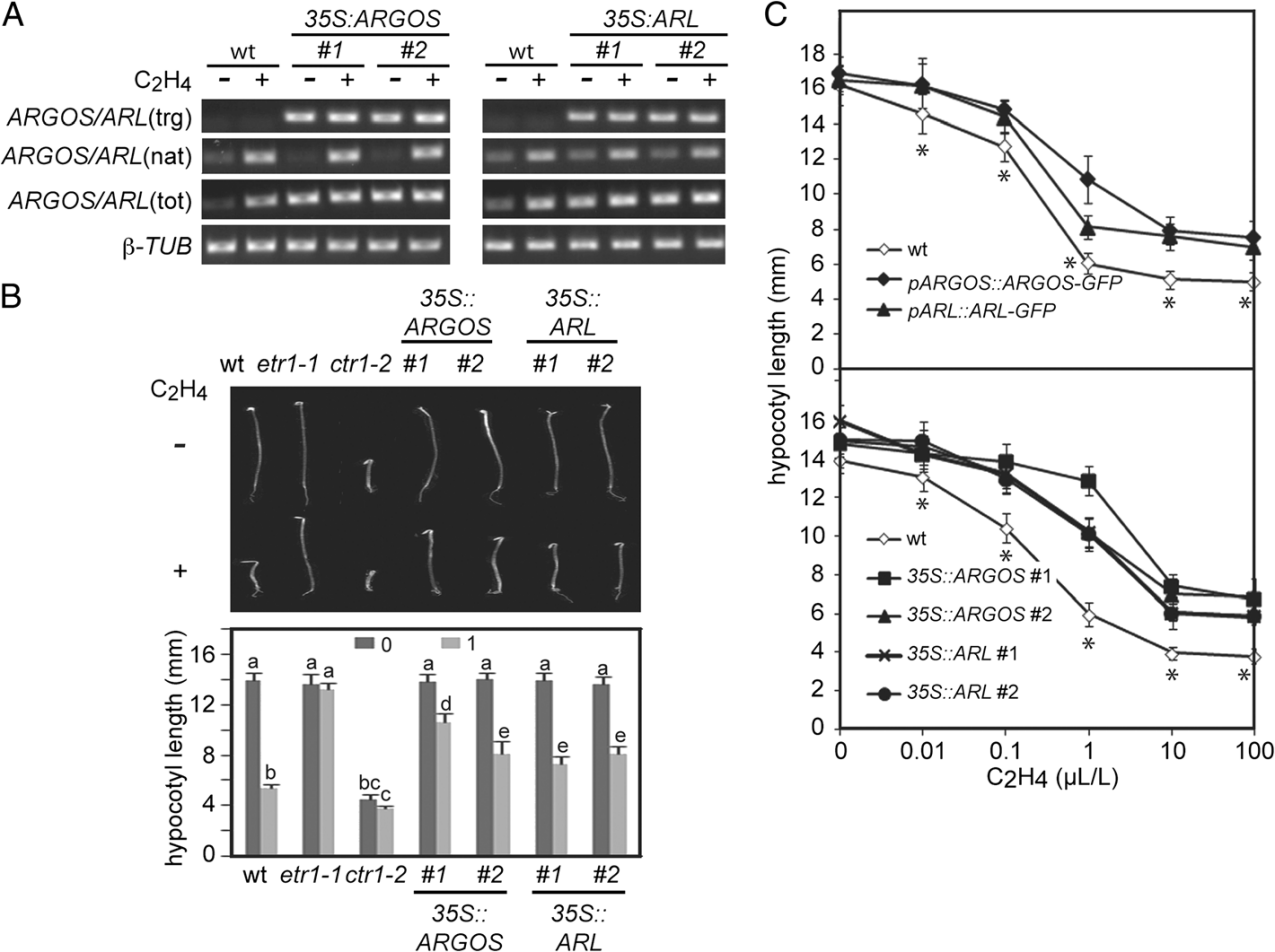
Functional analysis of the *ARGOS* and *ARL* in regulating ethylene sensitivity. **a** Increased expression levels of *ARGOS* in *CaMV 35S:ARGOS* lines and of *ARL* in *CaMV 35S:ARL* lines compared to wild type. Two independent transgenic lines (#1, #2) were analyzed for each construct, expression levels being examined in green seedlings following 2-h treatment in the absence or presence of 10 μL L^−1^ ethylene. β-tubulin served as a loading control. Primers were designed to specifically amplify the transgene (trg), the native gene (nat), or the total expression of native and transgene together (tot). **b** Phenotypic analysis of 4-day-old dark-grown seedlings grown in the absence or presence of 1 μL L^−1^ ethylene. Growth of transgenic lines were compared to the the ethylene-insensitive mutant *etr1-1* and the constitutive ethylene response mutant *ctr1-2*. Seedlings were examined for significant differences in growth based on the Tukey multiple range test among the means on the analysis of variance (p < 0.05; *n* = 10). Seedling measurements designated with the same letter exhibit no significant difference. Error bars indicate SD. **c** Ethylene dose response curves for hypocotyl growth in mutant lines compared to wild-type dark-grown seedlings. Error bars indicate SD (*n* = 10). Statistical significance of wild type to the transgenic lines was performed by an analysis of variance applying Bonferroni correction post-test comparisons (* p < 0.05)

We found that the *ARGOS:ARGOS-GFP* and *ARL:ARL-GFP* lines affected ethylene sensitivity based on the growth response of dark-grown seedlings to ethylene. Wild-type seedlings exhibit a pronounced reduction in hypocotyl growth when grown in 1 μL L^−1^ ethylene (Fig. 4B, C). Dose response analysis indicated that both the *ARGOS:ARGOS-GFP* and *ARL:ARL-GFP* exhibit reduced ethylene sensitivity (p < 0.05) compared to wild type at concentrations ranging from 0.01 to 100 μL L^−1^ ethylene (Fig. 4c). Since the *ARGOS:ARGOS-GFP* and *ARL:ARL-GFP* transgenes are expressed in the wild-type background, ethylene treatment will result in an overall heightened level of *ARGOS* family expression compared to wild type due to the presence of the native gene and the transgene. These data indicate that ARGOS and ARL can function as negative regulators of the ethylene response. Furthermore, the finding that such a phenotype can be induced from the native promoter is suggestive that changes in expression levels of *ARGOS* and *ARL* normally regulate ethylene sensitivity.

We tested the hypothesis that *ARGOS* and *ARL* function as negative regulators by generating transgenic lines overexpressing these genes under the constitutive *CaMV 35S* promoter. Overexpression lines exhibited increased basal expression of *ARGOS* or *ARL* in the absence of ethylene, and resulted in higher than wild-type expression in the presence of ethylene (Fig. 4a). The ethylene sensitivity of the *ARGOS* and *ARL* overexpression lines was significantly reduced compared to wild-type based on the dark-grown hypocotyl growth response to 1 μL L^−1^ ethylene (Fig. 4b). For comparison in this analysis, we also included the ethylene-insensitive mutant *etr1-1* which lacks this hypocotyl growth response to ethylene [33, 37], and the constitutive ethylene-response mutant *ctr1-2* which displays reduced hypocotyl growth in the both the absence and presence of ethylene (Fig. 4b). Dose response analysis demonstrating reduced sensitivity (p < 0.05) of the *ARGOS* and *ARL* overexpression lines compared to wild type from 0.01 to 100 μL L^−1^ ethylene (Fig. 4c).

### Overexpression of *ARGOS* and *ARL* recapitulates the effects on leaf cell expansion and division found in the ethylene-insensitive mutant *etr1-1*

**Fig. 5:**
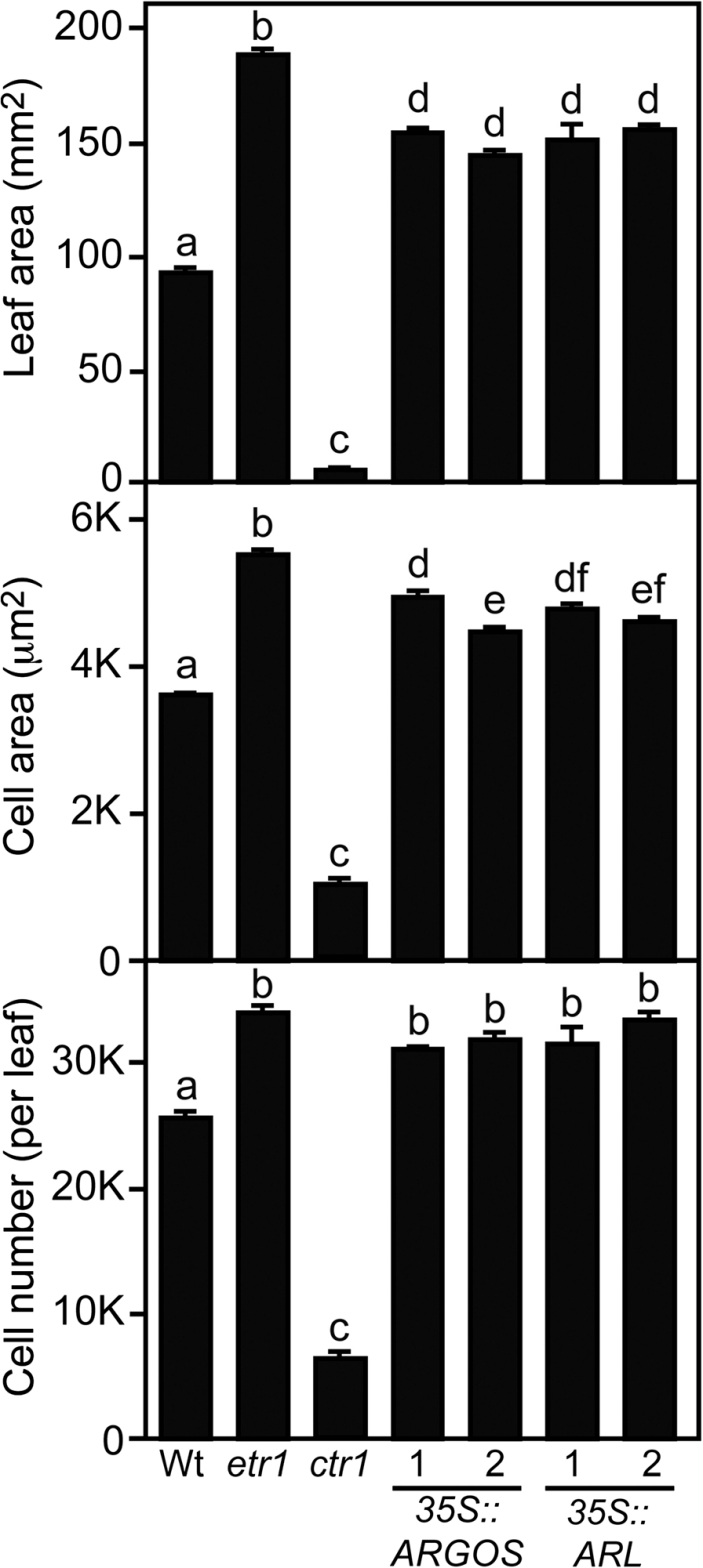
Overexpression of *ARGOS* and *ARL* recapitulate the effects on leaf cell expansion and division found in ethylene-insensitive mutants. Leaf area (*n* = 4), cell area (*n* = 40), and cells per leaf (*n* = 4) were determined for the fully expanded 5^th^ leaf of 30-day-old plants. Significant differences are based on the Tukey multiple range test among the means on the analysis of variance (p < 0.05); measurements designated with the same letter exhibit no significant difference. Error bars indicate SD

A defining characteristic for *ARGOS* family mutants is their effect on plant biomass, their overexpression resulting in increased leaf area, these effects being attributed to changes in cell expansion and/or cell proliferation [28–31]. Ethylene insensitivity results in a similar increase in leaf area [32, 37, 38], suggestive that effects of *ARGOS*-family overexpression on biomass could be due to altered ethylene signaling. To assess the role of the ethylene signal-transduction pathway in regulating cell expansion and cell proliferation, we characterized fully expanded leaves of the ethylene-insensitive mutant *etr1-1* [37] and the constitutive ethylene-response mutant *ctr1-2* [5]. As shown in Fig. 5, ethylene insensitivity results in increased leaf area due to an increase in both cell size and cell number. Conversely, the reduced leaf area found in *ctr1-2* arises from a decrease in both cell size and cell number. Overexpression of *ARGOS* or *ARL* results in increased leaf area, consistent with prior studies [28, 30], this change being due to a significant increase in both cell expansion and cell proliferation (Fig. 5). Thus the changes in leaf area arising from altered expression of the ARGOS family are consistent with what is observed in mutants that affect ethylene signaling.

### The *ARGOS* family regulates ethylene-dependent gene expression

**Fig. 6:**
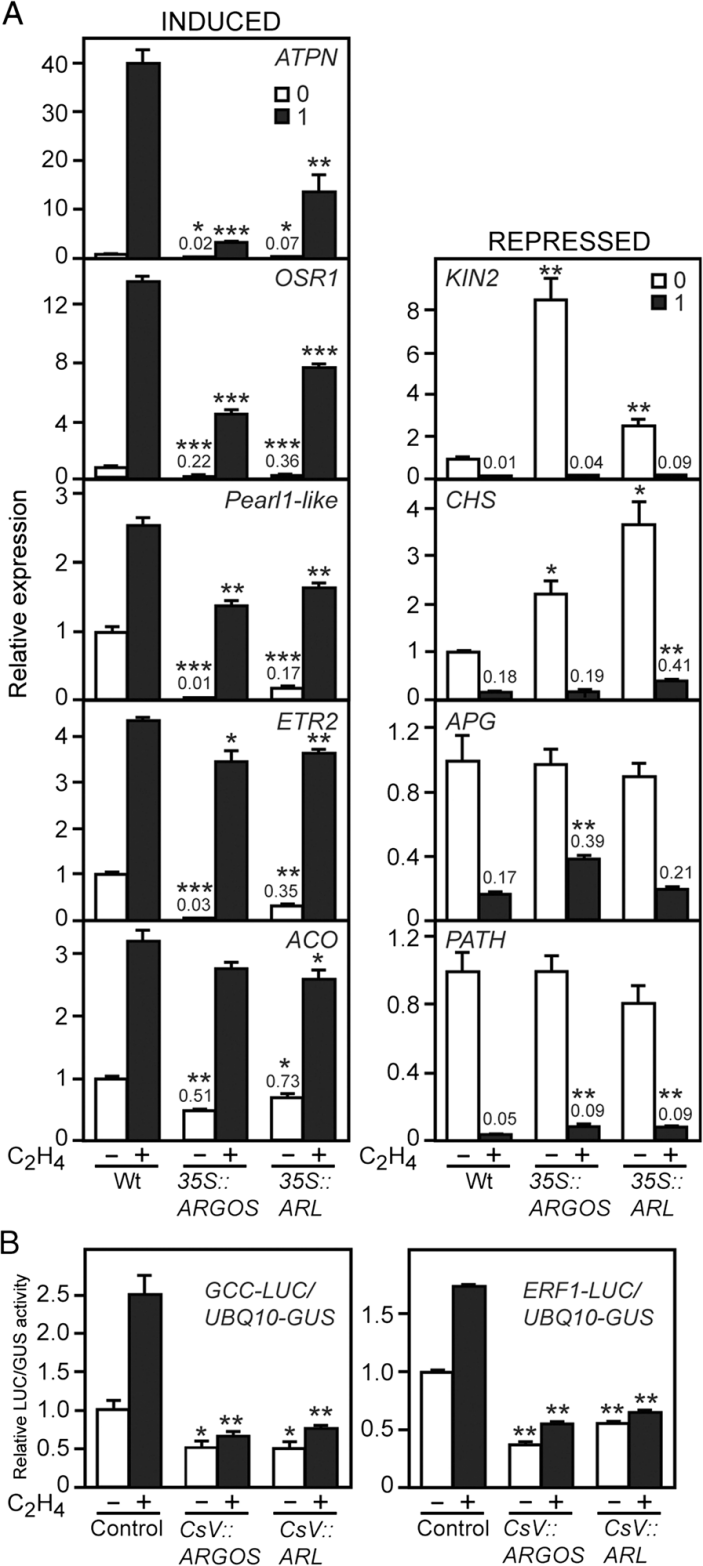
Overexpression of the *ARGOS* and *ARL* suppresses ethylene-dependent gene expression. **a** Gene expression was analyzed in dark-grown seedlings of the overexpression lines (*35S::ARGOS* #1 and *35S::ARL* #2) grown in the absence and presence of 1 μL L^−1^ ethylene. Expression of the indicated genes was determined by qRT-PCR, with the expression level of wild-type in the absence of ethylene set to 1. The left column shows genes whose expression is induced by ethylene, the right column genes whose expression is suppressed by ethylene. Data based on three biological replicates for each treatment. Statistical analysis was performed by unpaired two-tailed *t*-test with a Bonferroni correction (* p < 0.05; ** p < 0.01, *** p < 0.001), between the experimental samples and the corresponding wild-type control. **b** Overexpression of *ARGOS* or *ARL* reduces ethylene-dependent gene expression in a transient protoplast assay. Protoplasts were transfected with either of the ethylene-dependent luciferase reporters *GCC-LUC* or *ERF1-LUC*, as well as with a *UBQ-GUS* transgene for normalization of the transfection. Protoplasts were co-transfected with CsV::*ARGOS* or CsV::*ARL* constructs to determine their effect on the expression of the luciferase reporters. Protoplasts were treated in the absence or presence 10 μL L^−1^ ethylene for 6 h prior to determining the relative LUC/GUS activity. Data from one experiment is shown from two independent experiments with similar results, each experiment including two biological replicates per sample treatment. Statistical analysis was performed by *t*-test with a Bonferroni correction (* p < 0.05; ** p < 0.01), between the experimental samples and the corresponding wild-type control

To gain information at the molecular level as to how the ethylene response differs between wild type and the *ARGOS*-family overexpression lines, we examined ethylene-dependent gene expression (Fig. 6a). RNA was prepared from seedlings grown in the dark in the absence or presence of 1 μL L^−1^ ethylene, because we observed substantial differences in the hypocotyl growth response under these conditions (Fig. 4b, c). Five reporter genes we previously identified as robustly induced by ethylene [10, 39], were all induced in the wild-type seedlings (Fig. 6a). The molecular response to ethylene was altered in the *35S::ARGOS* and 35S::*ARL* lines (Fig. 6a). First, the basal expression of the reporter genes was reduced compared to wild type, indicating a reduced response to endogenous ethylene in the *35S::ARGOS* and *35S::ARL* lines. Second, the expression level of the reporter genes in response to 1 μL L^−1^ ethylene was also reduced in the transgenic lines compared to wild type. Thus overall, we observed consistently reduced expression for the reporter genes in the *35S::ARGOS* and *35S::ARL* lines.

We also examined the molecular response for four genes whose expression is repressed in response to 1 μL L^−1^ ethylene in wild type (Fig. 6a) [39]. Effects of the transgenic lines on the ethylene-repressed genes were less consistent than on the set of ethylene-induced genes. However, notably, the basal expression levels for both *KIN2* and *CHS* were significantly higher in the *35S::ARGOS* and *35S::ARL* lines (Fig. 6a), consistent with the response to endogenous ethylene in these lines being compromised such that they no longer suppress expression of these genes effectively.

As an alternative approach to examine the ability of *ARGOS* family members to inhibit ethylene-dependent gene expression, we employed a transient protoplast assay with the ethylene-inducible luciferase reporters *ERF1-LUC* or *GCC-LUC* [40–42]. Treatment of protoplasts with ethylene induces expression of the luciferase reporters (Fig. 6B). However, co-transfection with either *ARGOS* or *ARL* reduces the responsiveness of the luciferase reporter to the exogenous ethylene treatment. The basal level of expression for the luciferase reporter is also reduced, indicating that expression of *ARGOS* or *ARL* reduces the response to endogenous ethylene. Overall, these molecular results reveal that the *ARGOS* family members function as negative regulators of the ethylene-signaling pathway.

### The *ARGOS* family regulates the desensitization response of seedlings to ethylene

**Fig. 7:**
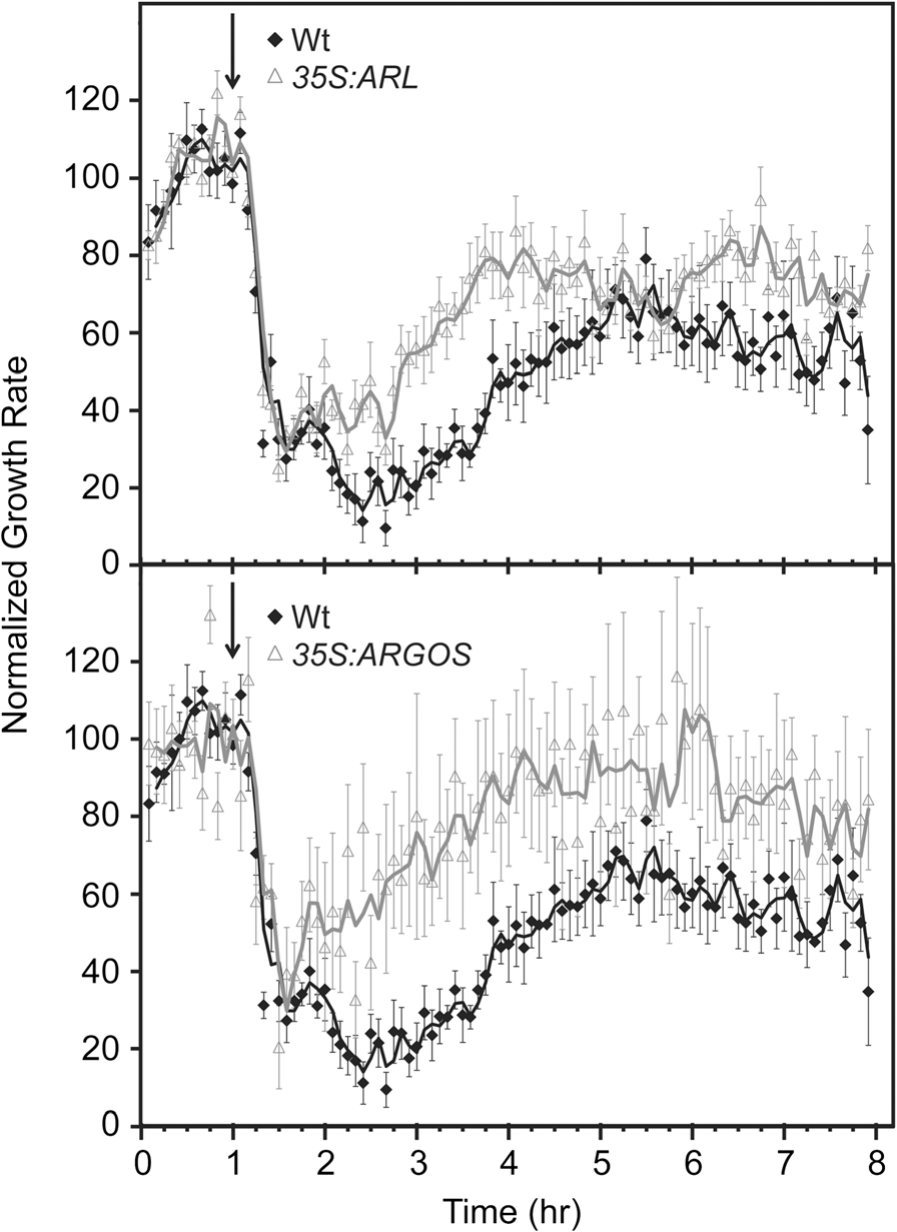
Overexpression of *ARGOS* and *ARL* accelerate the desensitization response to ethylene. Ethylene growth response kinetics were analyzed in dark-grown seedlings. Measurements were made in air for 1 h prior to introducing 0.1 μL L^−1^ ethylene (arrow), and growth rates normalized to the growth rate during this first hour. Error bars represent SE (*n* = 6 for wild-type, 8 for *35S::ARGOS*, 12 for *35S::ARL*)

We used time-lapse imaging to examine the growth inhibition kinetics of hypocotyls from two-day-old, dark-grown Arabidopsis seedlings. We have previously shown that application of higher concentrations of ethylene (>1 μL L^−1^) to wild-type Arabidopsis seedlings results in a rapid reduction in growth rate approximately 10 min after application of ethylene, with seedlings reaching a new steady state growth rate approximately 75 min after ethylene addition [9, 12]. Application of 0.1 μL L^−1^ ethylene causes growth inhibition with kinetics that are initially indistinguishable from higher dosages [9]. However, approximately 2.5 h after 0.1 μL L^−1^ ethylene application, desensitization is observed where the growth rate increases to a new steady-state rate approximately 50 % of that observed in air [9]. We obtained similar results with wild-type seedlings in the current study (Fig. 7). Overexpression of either *ARGOS* or *ARL* resulted in seedlings that initiated desensitization with a shorter delay and which reached a higher growth rate than wild-type seedlings (Fig. 7, Table 1). These results indicate that changes in expression of *ARGOS* family members modulate the seedling desensitization response to ethylene.

**Table 1:** Growth rates of seedlings during analysis of ethylene growth response kinetics

	Growth rate (mm h^−1^)^a^	
Seed line	Air^b^	Ethylene^c^
Wild-type	0.36 ± 0.01	0.20 ± 0.00
35S::ARGOS #1	0.32 ± 0.01	0.23 ± 0.01^*^
35S::ARL #2	0.34 ± 0.01	0.24 ± 0.00^*^

^*^Statistically different from wild-type, *P* < 0.01

^a^Average ± SEM

^b^Calculated from 1 h air pre-treatment

^c^Calculated from 4 to 7 h after ethylene added

## Discussion

Our results indicate that a key physiological role of the ARGOS family is to desensitize plants to ethylene. Desensitization is a common feature of sensory systems and is, for example, incorporated into such diverse systems as bacterial chemosensing, yeast osmosensing, and mammalian olfactory and light sensing [43]. Desensitization (or adaptation) is typified by the sensitivity to a signal being altered in response to changes in the level of the signal, thereby allowing the organism to sense the signal over as wide a range as possible. Arabidopsis can sense changes in ethylene concentration over six orders of magnitude [9, 10], consistent with a desensitization response. Short-term analysis of ethylene growth kinetics previously demonstrated that seedlings exhibit a desensitization response when treated with 0.01 μL L^−1^ ethylene, the timing of the response being consistent with the transcriptional induction of a negative regulator such as the *ARGOS* gene family [9], our data demonstrating that altered expression of *ARGOS* family members perturbs this desensitization response. Differing induction kinetics suggests that members of the *ARGOS* family could modulate the desensitization response across a range of ethylene concentrations. Ethylene induction of the ARGOS gene family is likely to be a primary ethylene response based on the presence of binding sites for the transcription factor EIN3 in the promoters of *ARGOS*, *ARL*, and *OSR2* [44].

Additional mechanisms that may allow for desensitization of the ethylene response have been identified. As with the *ARGOS* family, these involve transcriptional induction by ethylene of negative regulators. First, the ethylene receptors are negative regulators and several members are induced by ethylene (ERS1, ETR2, and ERS2 in Arabidopsis), such induction being observed in dicots and monocots [12, 45]. Second, members of the RTE1/GR family are also negative regulators of ethylene signaling [21, 22]. RTE1 is an ethylene-inducible transmembrane protein that interacts with the ethylene receptor ETR1 and appears to stabilize the receptor in the conformation normally observed in the absence of ethylene, thereby decreasing the receptor’s sensitivity to ethylene [22, 46, 47]. Third, acting to control the level of transcriptional output, the F-box protein EBF2 is an ethylene-induced negative regulator that targets the EIN3/EIL family of transcription factors for degradation [16, 48]. Fourth, transcriptional repressors of the ERF family are induced by ethylene and feedback to down-regulate the ethylene transcriptional response [49, 50]. Interestingly, co-expression analysis places *ARGOS*, *ARL*, and *OSR1* in a network involving genes for the ethylene receptors ERS1 and ETR2 as well as for the F-box protein EBF2 [29], consistent with a role in regulating the ethylene response. The range of mechanisms for desensitization would allow for concerted regulation of ethylene responses from receptor to gene expression, and also provide multiple points for cross-talk with other pathways.

The mechanism by which the ARGOS family regulates ethylene signaling remains to be elucidated, but several predictions can be made. First, the mechanism is likely to involve the transmembrane portion of the ARGOS family proteins, the two transmembrane domains and the proline-rich linker joining them exhibiting the greatest sequence conservation, deletion analysis confirming that this region is sufficient to induce organ growth when overexpressed [29]. Second, the effect of the ARGOS family is likely to involve initial components of the ethylene signaling pathway based on their co-localization to the ER [4]. Third, the ARGOS family is likely to regulate signal output from a primary rather than a peripheral element of the pathway, because modulation of *ARGOS* family expression has broad effects on the physiological and molecular response to ethylene. Based on these characteristics, the ethylene receptors and EIN2 represent potential targets for regulation by the ARGOS family.

Based on our analysis, the previously described effects of *ARGOS* family mutants on plant biomass, occurring due to alterations in cell expansion and proliferation [28–30], can be ascribed to a role in modulation of the ethylene signaling pathway. Ethylene mutants are known to affect plant growth, ethylene insensitivity resulting in increased plant biomass [37, 51, 52], enhancement of ethylene signaling resulting in decreased biomass [5, 53]. Indeed, approaches to inhibit ethylene signaling have been pursued as a means to increase plant biomass under common environmental-stress conditions [54, 55]. Our results making use of ethylene pathway mutants support a role for ethylene in regulation of both cell expansion and proliferation in Arabidopsis leaves, these effects both contributing to changes in biomass, consistent with the effects we observed in *ARGOS* family mutants. We note that prior characterization of *ARGOS*-family overexpression lines indicated effects on cell proliferation for *ARGOS* [28], cell expansion for *ARL* and *OSR2* [30, 31], and both for *OSR1* [29]. Our results indicate that *ARGOS* and *ARL*, like *OSR1*, regulate both cell expansion and proliferation. Differences in ascribing roles in cell proliferation and/or expansion can arise due to such factors as to the leaf chosen for analysis and whether the leaf has reached full expansion, prior interpretation for the effects of ethylene mutants on leaf area having differed due to such variables [37, 56].

The ARGOS family is plant-specific and the role it plays in modulating plant biomass has led to its characterization in other plant species, including the dicot cabbage and the monocots rice and maize. Overexpression of an *ARGOS* homologue from cabbage (*BrARGOS*) in Arabidopsis resulted in increased organ growth, with an increase in leaf area arising primarily due to an increase in cell proliferation [57]. Similarly, overexpression of the rice homologue *OsARGOS* in Arabidopsis also resulted in increased organ growth, although here the increase in leaf area was attributed to effects on both cell proliferation and expansion [58]. Interestingly, no effects on organ size were found when *OsARGOS* was overexpressed in rice. However the effects of ethylene insensitivity on the adult rice phenotypes examined are subtle [59] and so the lack of a phenotype is consistent with what might be expected based on a role for *OsARGOS* in ethylene signaling. It will be of interest to determine if the transgenic rice lines have altered ethylene-related phenotypes for root and coleoptile growth, senescence, and grain weight per plant. The maize homologue to *ARGOS* (*ZmZAR1*) has also been characterized, its transgenic overexpression in maize resulting in increased plant and organ growth, including that of leaves, stalks, and ears [60]. Increased leaf area of the *ZmZAR1* overexpressing lines was primarily due to an increase in cell proliferation rather than cell expansion. Overexpression of *ZmZAR1* was also implicated in an enhanced resistance to drought stress, a phenotype that could relate to a role in ethylene signaling because a reduction in ethylene activity enhances drought tolerance in maize [61].

Of interest is our finding that ethylene pathway mutants affect cell proliferation as well as cell expansion. Although the role of ethylene as an inhibitor of cell expansion is well documented in Arabidopsis, a role for ethylene in the control of cell proliferation is more novel. However, earlier data from agronomic plant species as well as recent data from Arabidopsis support an inhibitory role for ethylene in cell division [1, 62]. In particular, treatment of Arabidopsis seedlings with the ethylene precursor ACC inhibited cell proliferation in leaves as well as activity of cyclin-dependent kinase A [62]. Our analysis of ethylene-pathway mutants is consistent with an inhibitory role for ethylene in the regulation of cell proliferation. The five-fold difference in leaf cell number we observe between the ethylene-insensitive mutant *etr1-1* and the constitutive ethylene response mutant *ctr1-2* support a substantive contribution of ethylene to the regulation of cell proliferation.

## Conclusions

**Fig. 8:**
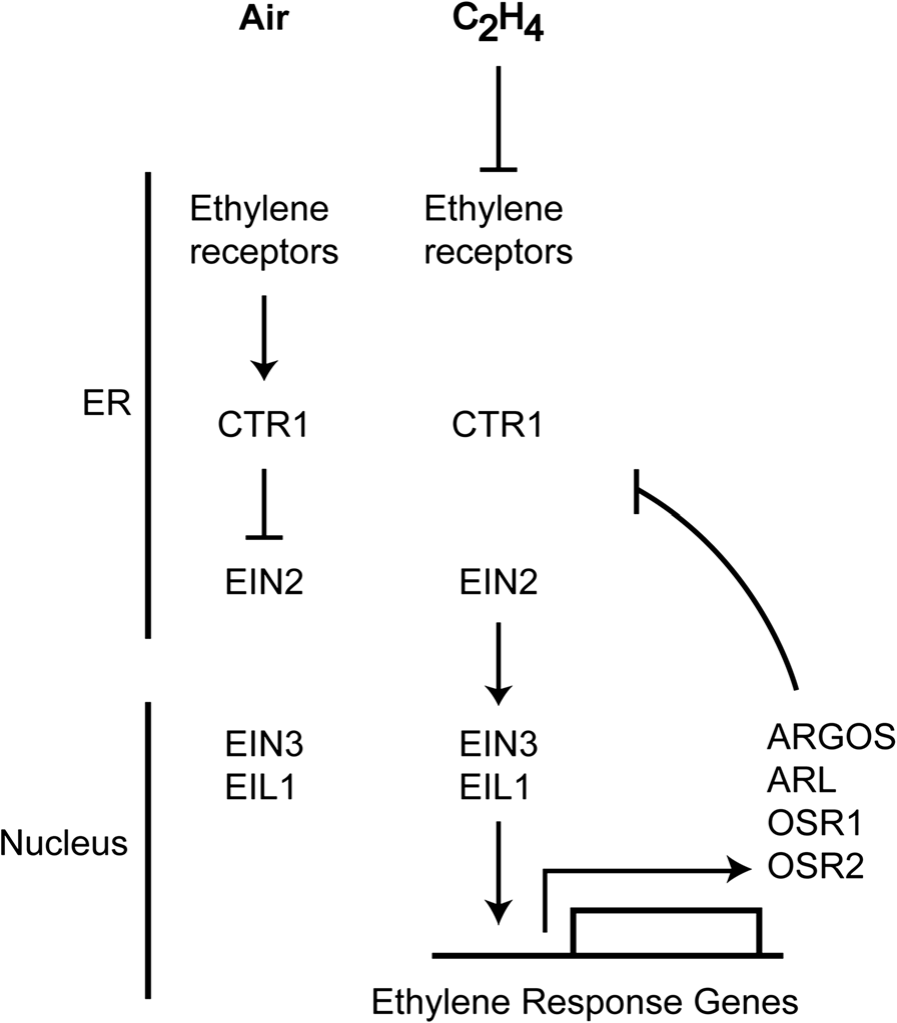
Model for negative feedback loop by which the ARGOS family desensitizes ethylene signaling. Key signaling elements of the ethylene pathway are shown, initial elements being localized to the endoplasmic reticulum (ER), the EIN3 transcription factor family being localized to the nucleus. Ethylene activates the transcriptional response to ethylene, stimulating expression of *ARGOS* family members. ARGOS family proteins insert into the ER where they serve to desensitize the ethylene signal output

We demonstrate a new role for the *ARGOS* gene family in negatively regulating the ethylene response of Arabidopsis. A model integrating the ARGOS family with the ethylene signaling pathway is shown in Fig. 8. Members of the *ARGOS* family are transcriptionally induced in response to ethylene, induction requiring signaling through the primary ethylene signaling pathway. ARGOS and ARL are targeted to the endoplasmic reticulum, the same subcellular location as initial signaling elements in the ethylene pathway. Consistent with their role as negative regulators of the ethylene pathway are the effects of overexpression lines on (1) the triple response of dark-grown seedlings to ethylene, (2) the short-term kinetic response to ethylene, (3) cell expansion and proliferation in leaves, and (4) ethylene-dependent gene expression. Together these data support a model in which the ARGOS gene family functions as part of a negative feedback circuit to desensitize the plant to ethylene, thereby expanding the range of ethylene concentrations to which the plant can respond. These results also indicate that the effects of the *ARGOS* gene family on plant growth and biomass are mediated through effects on ethylene signal transduction.

## Methods

### Constructs and plant transformation

To make constructs for expression of *ARGOS* (At3g59900) and *ARL* (At2g44080) with C-terminal GFP tags and driven under their native promoters, genomic fragments including 5′ flanking regions (1640 bp and 1479 bp for *ARGOS* and *ARL*, respectively) and coding regions (390 bp and 405 bp for *ARGOS* and *ARL*, respectively) were amplified from wild-type Col-0 genomic DNA, using the *ARGOS* primers 5′-TGGTCAACGATTCAAGGAGATCCA-3′ and 5′-GCCGATTGACATGAAATTGCAAGTTACATCTG-3′, and the *ARL* primers 5′-TAGCCACCACATGAAATGCCGAGA-3′ and 5′-GCCGATTGACATGAAATTGCAAGTTACATCTG-3′. Fragments were cloned into the entry vector pCR8 (Invitrogen, USA) and then recombined into the vector pGWB204 [63] using the Gateway system.

For overexpression of *ARGOS* and *ARL* in plants and protoplasts, their coding regions were amplified from cDNA, with *ARGOS* amplified using the primers 5′-GAATCCATGATTCGAGAAATCTCAAACTTAC-3′ and 5′-GGATCCTGACATGAAATTGCAAGTTACATCTG-3′ and *ARL* amplified using the primers 5′-GAATCATGATTCGTGAGTTCTCCAGTCTAC-3′ and 5′-GGATCCCATAAAAGTGGAAGAAGAAGAAACATG-3′, and the fragments cloned into pCR8. The fragments were moved into pEarleyGate100 [64] for stable plant transformation and into CsVGFP-999 [65] for protoplast transfection. For plant transformation, constructs were introduced into *Agrobacterium tumefaciens* strain GV3101 and transformed into Arabidopsis by the floral-dip method [66].

### Transient expression in *Arabidopsis* protoplasts

*Arabidopsis* protoplasts were isolated and transfected as described [67]. Visualization for localization studies was with a Leica TCS SP UV confocal microscope, GFP being imaged with a 488 nm laser, RFP with a 561 nm laser, and protoplasts with DIC optics. For analysis of the ethylene response, the reporter constructs *GCC-LUC* [40] and *ERF1-LUC* [42] were used. The *UBQ10-GUS* construct was used as an internal control. Transfected protoplast samples in culture dishes were placed in air-tight containers with or without 10 μL L^−1^ ethylene for 6 h at 22 °C under dim light (5 μE⋅m^−2^⋅s^−1^). The results are shown as the means of relative LUC activities from duplicate samples with error bars.

### Membrane fractionation and immunoblot analysis

Microsomal and soluble fractions were isolated from green *Arabidopsis* seedlings as described [36]. Briefly, plant material was homogenized in 30 mM Tris (pH 8), 150 mM NaCl, 10 mM EDTA, and 20 % (v/v) glycerol, with protease inhibitors cocktail (Sigma) and then centrifuged at 8,000 g for 15 min. The supernatant was then centrifuged at 100,000 g for 30 min, and the resulting membrane pellet was resuspended in 10 mM Tris (pH 7.6), 150 mM NaCl, 1 mM EDTA, and 10 % (v/v) glycerol with protease inhibitors cocktail.

Immunoblot analysis was performed as described [36]. The BCA assay (Pierce) was used to measure protein concentration as described [68]. Before SDS-PAGE, protein samples were mixed with SDS-PAGE loading buffer and incubated at 37 °C for 1 h to denature integral membrane proteins without aggregation. Primary antibodies used were HRP-conjugated monoclonal anti-GFP (Santa Cruz Biotechnology), anti-ETR1 [69], anti-BIP (Stressgen Biotech), and anti-ACC oxidase (Santra Cruz Biotechnology).

### Analysis of the ethylene response

For short-term ethylene treatment of green seedlings, seedlings were grown at 22 °C for two to three weeks with constant light on Murashige and Skoog basal medium with Gamborg’s vitamins (pH 5.75; Sigma), 1 % (w/v) sucrose and 8 % (w/v) agar [53], then treated for the indicated times and ethylene concentrations in sealed containers. Treatment and analysis of the triple response of dark-grown *Arabidopsis* seedlings to ethylene was performed as described [39]. Aminoethoxyvinylglycine (AVG; 5 μM), an inhibitor of ethylene biosynthesis, was included in the media to reduce endogenous ethylene production. For short-term kinetic analysis of dark-grown seedlings, time-lapse imaging and growth rate analysis of hypocotyls were carried out as previously described [9, 12]. To determine leaf size, cell area, and cells per leaf of ethylene pathway mutants compared to ARGOS family mutants, the fully expanded fifth leaf was analyzed from 30-day-old plants grown under an 18-h light/6-h dark cycle. Cell area was determined from palisade cells at the central region of the leaf beside the mid-vein as described [29], with 10 palisade cells characterized per leaf. Area determination measurements were made with IMAGE J software (http://rsbweb.nih.gov/ij/). ANOVA tables were generated using http://www.physics.csbsju.edu/stats/anova.html and multiple comparison tests done using http://graphpad.com/quickcalcs/posttest1/ quick calc web tool that uses the Bonferroni correction for post-test comparisons.

### Quantitative real-time PCR and RT-PCR

Total RNA was extracted from seedlings using the RNeasy Plant Mini Kit (Qiagen, USA) and cDNA synthesized using the First Strand cDNA Synthesis Kit (Invitrogen, USA) as described [70]. For quantitative RT-PCR, RNA from three biological replicates was used as template for first-strand cDNA synthesis, and three experimental replicate reactions were performed for each biological replicate using primer pairs specific for the genes of interest. The following primers were used: *ARGOS* (5′-GTCATGGACGTCGGAAGAAACAAC-3′ and 5′-GGGAACCAATAGCAGCATAAACGG-3′); *ARL* (5′-CAACAACAACATGGACGTGAGAGG-3′ and 5′- GGAGGCAATGGTGGAAGAATCAAC-3′); *OSR1* (At2g41230) (5′-ATGAGGGTTCATGATCAACGGCTG-3′ and 5′-GGCTGGGCTCATTAGAAGGAGAAA-3′); *OSR2* (At2g41225) (5′-TGATGGTGCTATTGGCGGTT-3′ and 5′- CAAACGACGACGCATTCACA-3′), *ERS1* (At2g40940) (5′-ACCTATGTGTGCAGGTGAAGGACA-3′ and 5′-AGCCCGACAAACCGTTTACAG AGA-3′); *ERS2* (At1g04310) (5′-TCAAGAAGCGGTTTGGCTACATTG-3′ and 5′-TAGACCGTCCTCAACAACCCGAAT-3′); *ETR2* (At3g23150) (5′-AGAGAAACTCGGGTGCGATGT-3′ and 5′-TCACTGTCGTCGCCACAATC-3′); Peroxidase ATP-N (At5g19890) (5′-AGTGACTTAGCCGTGAACACCACA-3′ and 5′-ACGAGACCGATCAACTCCCAAACA-3′); *ACO* ACC-oxidase (At1g77330) (5′-GTGATGGATGAGAATTTGGGTTTGCC-3′ and 5′-ATCGATCCACTCGCCGTCTTTCAA-3′); and pEARL1-like (At4g12470) (5′-AGTCCTAAACCAAAGCCAGTCCCA-3′ and 5′-CGATATTGTGCACTGGCATCGCAT-3′), *KIN2* (AT5G15960) (5′-TGTATCGGATGCGGCAGCG-3′ and 5′-TTTGAATATAAGTTTGGCTCGTCT-3′), *CHS* (At5g13930) (5′-TGCTTACATGGCTCCTTCTCTGGA-3′ and 5′-ATCTCAGAGCAGACAACGAGGACA-3′), APG-like (AT1G75900) (5′-TTTGCGTCCGGAGGTTCTGGTTAT-3′ and 5′-CTGAGGCAGAGTCAGACATAAGAG-3′), and a pathogen-related gene (AT4G25780) (5′-TGACCACGACTCCTTGCAGTTCTT-3′ and 5′-ATGAAGATCCCACCATTGTCGCAC-3′). For RT-PCR, the ARGOS transgene (5′-GGTCTAACGGCATCTCTGTTAAT-3′ and 5′-GTACAAGAAAGCTGGGTCGAA-3′) and the native gene (5′-CCAGTTGCCCTAAAGATCAG-3′ and 5′-GTCCATGACTCGGTTGTTC-3′) were specifically amplified with the indicated primers; the ARL transgene (5′-TGTTGGTCTCACAGCATCTC-3′ and 5′-CACCTAGGCACCACTTTGTA-3′) and the native gene (5′-CTCAAGTTTCTTCTTCATACATCG-3′ and 5′-TCCGGTTATGATCTCCTCTC-3′) were specifically amplified with the indicated primers; total expression levels were determined with the primers listed earlier for qRT-PCR. *Beta-tubulin* (At5g62700) was used as a control for qRT-PCR with primers 5′-CGTAAGCTTGCTGTGAATCTCATC-3′ and 5′-CTGCTCGTCAACTTCCTTTGTG-3′ and for RT-PCR with primers 5′-TGGTGGAGCCTTACAACGCTACTT-3′ and 5′-TTCACAGCAAGCTTACGGAGGTCA-3′. For RT-PCR of the

## Additional files

Additional file 1:
**Characteristics of the ARGOS family.** Arabidopsis contains a four-member gene family encoding AtARGOS, AtARL, AtOSR1, and AtOSR2. The moss *Physcomitrella patens* has a gene encoding a related protein (PpARL). At indicates Arabidopsis; Pp indicates *Physcomitrella patens*. (A) Amino acid sequence alignment. Two predicted transmembrane domains are highlighted in yellow. Residues identical to the consensus (Majority) are highlighted. Note the highly conserved proline-rich region at the turn between the two predicted transmembrane domains. Clustal alignment was performed on the multiple sequences using the Lasergene MegAlign program (DNASTAR, Inc.). (B) Phylogenetic relationship derived from the multiple sequence alignment. Units indicate number of substitution events. Additional file 2:
**Hormonal induction of the ARGOS gene family based on microarray analysis.** Three-dimensional graphs are shown for expression of *ARGOS*, *ARL*, and *OSR1*, with hormone treatment on the X-axis, relative expression on the Y-axis, and time on the Z-axis. Data was extracted from AtGEnExpress using the Weigelworld interface (http://www.weigelworld.org/resources/microarray/AtGenExpress) for green seedlings treated for 0.5 (blue), 1 (red), and 3 (yellow) hours with a water control (H_2_O), the ethylene biosynthetic precursor aminocyclopropane-carboxylic acid (ACC), auxin (IAA), gibberellic acid (GA), abscisic acid (ABA), and cytokinin (zeatin).

## Abbreviations

ACC: 1-aminocyclopropane-1-carboxylic acid
AVG: Aminoethoxyvinylglycine
ER: Endoplasmic reticulum
GFP: Green-fluorescent protein
IAA: Indole-3-acetic acid
NAA: 1-naphthaleneacetic acid
