# Supplementary figures and images for: The ARGOS gene family functions in a negative feedback loop to desensitize plants to ethylene
Source: BMC Plant Biol. 2015 Jun 24;15:157. doi: 10.1186/s12870-015-0554-x (PMC4478640; doi:10.1186/s12870-015-0554-x)

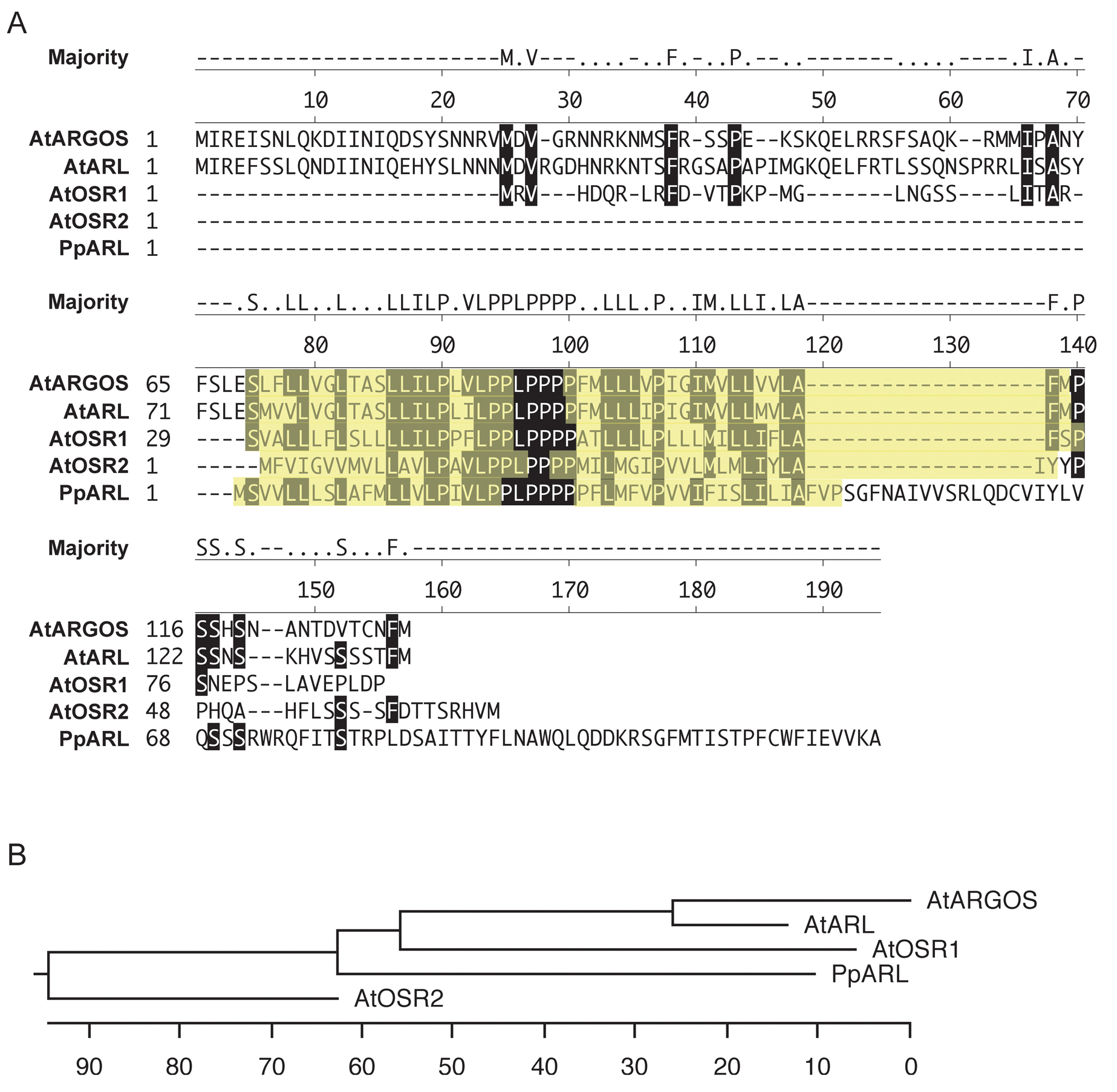

Supplement: Additional file 1: — Characteristics of the ARGOS family. Arabidopsis contains a four-member gene family encoding AtARGOS, AtARL, AtOSR1, and AtOSR2. The moss Physcomitrella patens has a gene encoding a related protein (PpARL). At indicates Arabidopsis; Pp indicates Physcomitrella patens. (A) Amino acid sequence alignment. Two predicted transmembrane domains are highlighted in yellow. Residues identical to the consensus (Majority) are highlighted. Note the highly conserved proline-rich region at the turn between the two predicted transmembrane domains. Clustal alignment was performed on the multiple sequences using the Lasergene MegAlign program (DNASTAR, Inc.). (B) Phylogenetic relationship derived from the multiple sequence alignment. Units indicate number of substitution events. [file 12870_2015_554_MOESM1_ESM.jpeg]

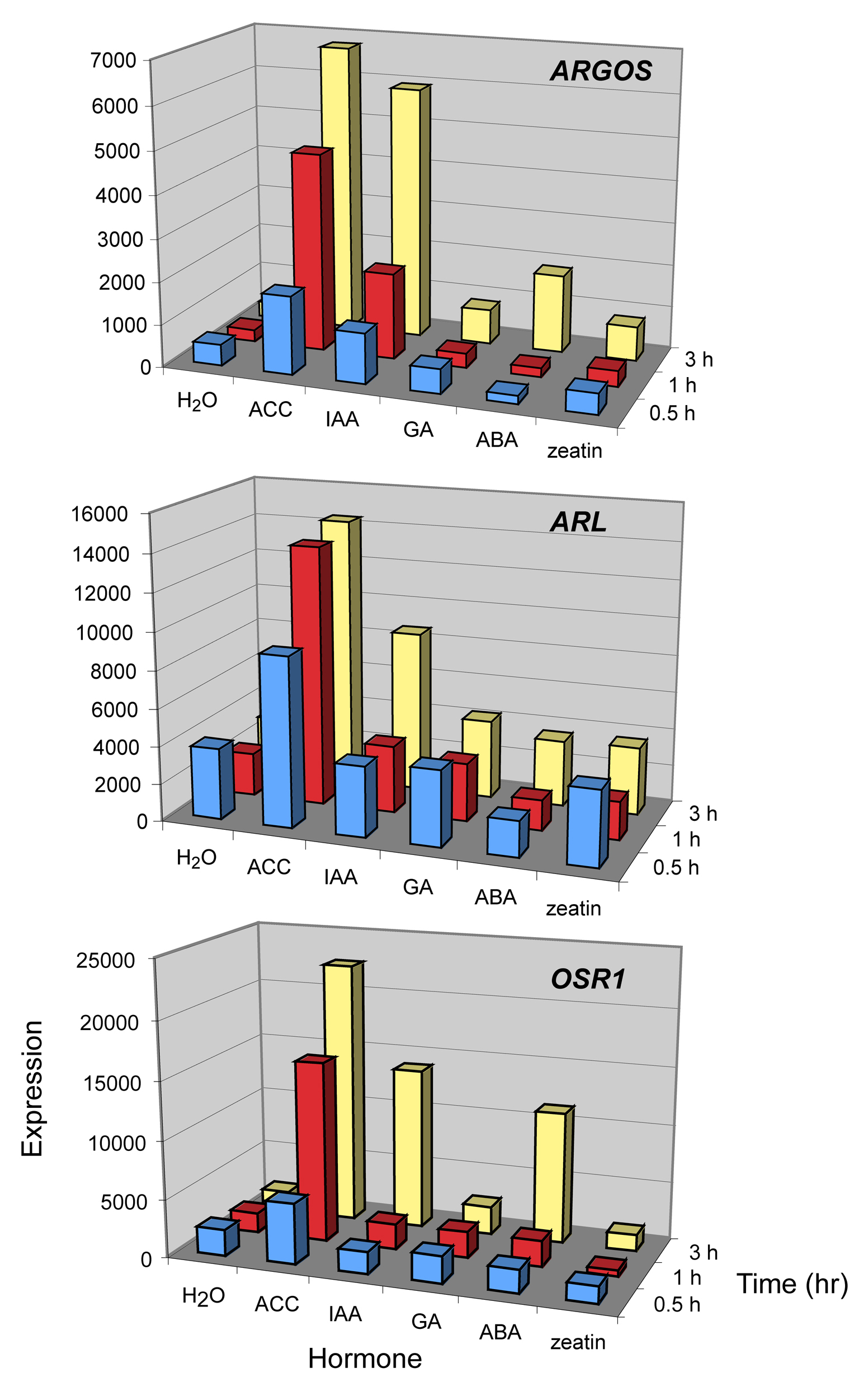

Supplement: Additional file 2: — Hormonal induction of the ARGOS gene family based on microarray analysis. Three-dimensional graphs are shown for expression of ARGOS, ARL, and OSR1, with hormone treatment on the X-axis, relative expression on the Y-axis, and time on the Z-axis. Data was extracted from AtGEnExpress using the Weigelworld interface (http://www.weigelworld.org/resources/microarray/AtGenExpress) for green seedlings treated for 0.5 (blue), 1 (red), and 3 (yellow) hours with a water control (H2O), the ethylene biosynthetic precursor aminocyclopropane-carboxylic acid (ACC), auxin (IAA), gibberellic acid (GA), abscisic acid (ABA), and cytokinin (zeatin). [file 12870_2015_554_MOESM2_ESM.jpeg]
